# Bacterial factors required for *Streptococcus pneumoniae* coinfection with influenza A virus

**DOI:** 10.1186/s12929-021-00756-0

**Published:** 2021-08-27

**Authors:** Yi-Yin Chen, Ching-Tai Huang, Shiao-Wen Li, Yi-Jiun Pan, Tzu-Lung Lin, Ya-Yu Huang, Ting-Hsuan Li, Yu-Ching Yang, Yu-Nong Gong, Yu-Chia Hsieh

**Affiliations:** 1 Department of Pediatrics, Chang Gung Children’s Hospital, Chang Gung Memorial Hospital, College of Medicine, Chang Gung University, Taoyuan, Taiwan; 2 Division of Infectious Diseases, Department of Internal Medicine, Chang Gung Memorial Hospital, Taipei, Taoyuan Taiwan; 3 Molecular Medicine Research Center, Chang Gung University, Taoyuan, Taiwan; 4 Department of Microbiology and Immunology, School of Medicine, College of Medicine, China Medical University, Taichung, Taiwan; 5 Department of Medical Biotechnology and Laboratory Science, College of Medicine, Chang Gung University, Taoyuan, Taiwan; 6 Research Center for Emerging Viral Infections, Chang Gung University, Taoyuan, Taiwan; 7 Department of Laboratory Medicine, Linkou Chang Gung Memorial Hospital, Taoyuan, Taiwan; 8 Department of Pediatrics, Linkou Chang Gung Memorial Hospital, No. 5, Fuxing Street, Guishan District, Taoyuan City, 333 Taiwan

**Keywords:** Influenza A, *S. pneumoniae*, Transposon mutant library, Coinfection, Metabolome

## Abstract

**Background:**

*Streptococcus pneumoniae* is a common cause of post-influenza secondary bacterial infection, which results in excessive morbidity and mortality. Although 13-valent pneumococcal conjugate vaccine (PCV13) vaccination programs have decreased the incidence of pneumococcal pneumonia, PCV13 failed to prevent serotype 3 pneumococcal disease as effectively as other vaccine serotypes. We aimed to investigate the mechanisms underlying the co-pathogenesis of influenza virus and serotype 3 pneumococci.

**Methods:**

We carried out a genome-wide screening of a serotype 3 *S. pneumoniae* transposon insertion mutant library in a mouse model of coinfection with influenza A virus (IAV) to identify the bacterial factors required for this synergism.

**Results:**

Direct, high-throughput sequencing of transposon insertion sites identified 24 genes required for both coinfection and bacterial infection alone. Targeted deletion of the putative aminotransferase (*PA*) gene decreased bacterial growth, which was restored by supplementation with methionine. The bacterial burden in a coinfection with the *PA* gene deletion mutant and IAV in the lung was lower than that in a coinfection with wild-type pneumococcus and IAV, but was significantly higher than that in an infection with the *PA* gene deletion mutant alone. These data suggest that IAV infection alters host metabolism to benefit pneumococcal fitness and confer higher susceptibility to pneumococcal infection. We further demonstrated that bacterial growth was increased by supplementation with methionine or IAV-infected mouse lung homogenates.

**Conclusions:**

The data indicates that modulation of host metabolism during IAV infection may serve as a potential therapeutic intervention against secondary bacterial infections caused by serotype 3 pneumococci during IAV outbreaks in the future.

**Supplementary Information:**

The online version contains supplementary material available at 10.1186/s12929-021-00756-0.

## Introduction

Influenza A virus (IAV) infection dramatically increases the susceptibility to secondary *Streptococcus pneumoniae* infections, resulting in significantly greater morbidity and mortality during IAV outbreaks [1]. In the most recent influenza pandemic caused by a triple-reassortment IAV subtype H1N1 occurred in 2009, bacterial pneumonia complicated between 25 and 50% of the severe infections, in both children and adults [2–4]. *S. pneumoniae* remained one of the important complicating organisms [5]. *S. pneumoniae* is a common human nasopharyngeal commensal that colonizes 10–40% of children under the age of 5 years [6] and 8–15% of adults [7]. For progression from colonization to lower respiratory tract infection, pneumococci must overcome physical barriers and escape immune defense mechanisms. Influenza virus disrupts lung physiology to promote pneumococcal adherence and interferes with host immune responses to facilitate secondary pneumococcal invasion [8, 9]. Furthermore, influenza neuraminidases remove terminal sialic acid residues from host glycoconjugates to promote pneumococcal adherence and growth during coinfection [10, 11]. On the pneumococcal side, the bacterial mechanism underlying this process remain largely unknown. It has been reported that virulence factors, such as pneumococcal surface protein A (PsaP), choline-binding protein A (CbpA), and pneumococcal serine-rich repeat protein (PsrP), can be used for increased adherence to the basement membrane or elements of the extracellular matrix, such as fibrin, fibrinogen, and collagen, in the presence of influenza [12]. Until now, there have been no systemic studies to identify genes of *S. pneumoniae* involved in IAV coinfection.

Development of a 13-valent pneumococcal conjugate vaccine (PCV13) has substantially reduced the global burden of pneumococcal disease [13]. Nevertheless, PCV13 failed to effectively prevent serotype 3 mucosal disease as other vaccine serotypes, which is associated with a higher risk of death [14, 15]. In the present study, a clinical isolate of serotype 3 (Taian-S3), recovered from a child with severe pneumonia [16], was used to construct a mini-mariner transposon library [17]. Using high-throughput sequencing to sequence the transposon insertion site can determine the relative fitness of bacterial mutants in the presence and absence of prior IAV infection for comprehensive understanding the interaction between IAV and pneumococcus [17, 18]. Here, we report a genome-wide profiling of the *S. pneumoniae* genes contributing to strong fitness benefits during coinfection with IAV in comparison with bacterial infection alone in mice.

## Materials and methods

### *S. pneumoniae* and influenza A virus culture conditions

Pneumococcal isolates Taian-S3 were grown at 37 °C in Todd–Hewitt broth supplemented with 0.5% yeast extract (THY), chemically defined medium (CDM) [19] or cultured on blood agar plate (supplemented with 5% defibrinated sheep blood) in the presence of 5% CO_2_. *Escherichia coli* (*E. coli*) was grown in Luria broth (LB). Antibiotics were added at the following concentrations, when required: spectinomycin at 250 mg/L for *S. pneumoniae* and at 100 mg/L for *E. coli*; ampicillin at 100 mg/L for *E. coli*; chloramphenicol at 10 mg/L for *S. pneumoniae* and at 30 mg/L for *E. coli.* The A/PR/8/34 (PR8) strain of influenza virus were grown in Madin-Darby Canine Kidney (MDCK) cells.

### Mutant library construction

A mutant library of the Tain-S3 strain was constructed using mini-transposon magellan6 random insertional mutagenesis as previously described [17]. Please see Additional file 1 for more detail methods.

### *S. pneumoniae* coinfection post influenza A virus infection in a mouse model

**Fig. 1:**
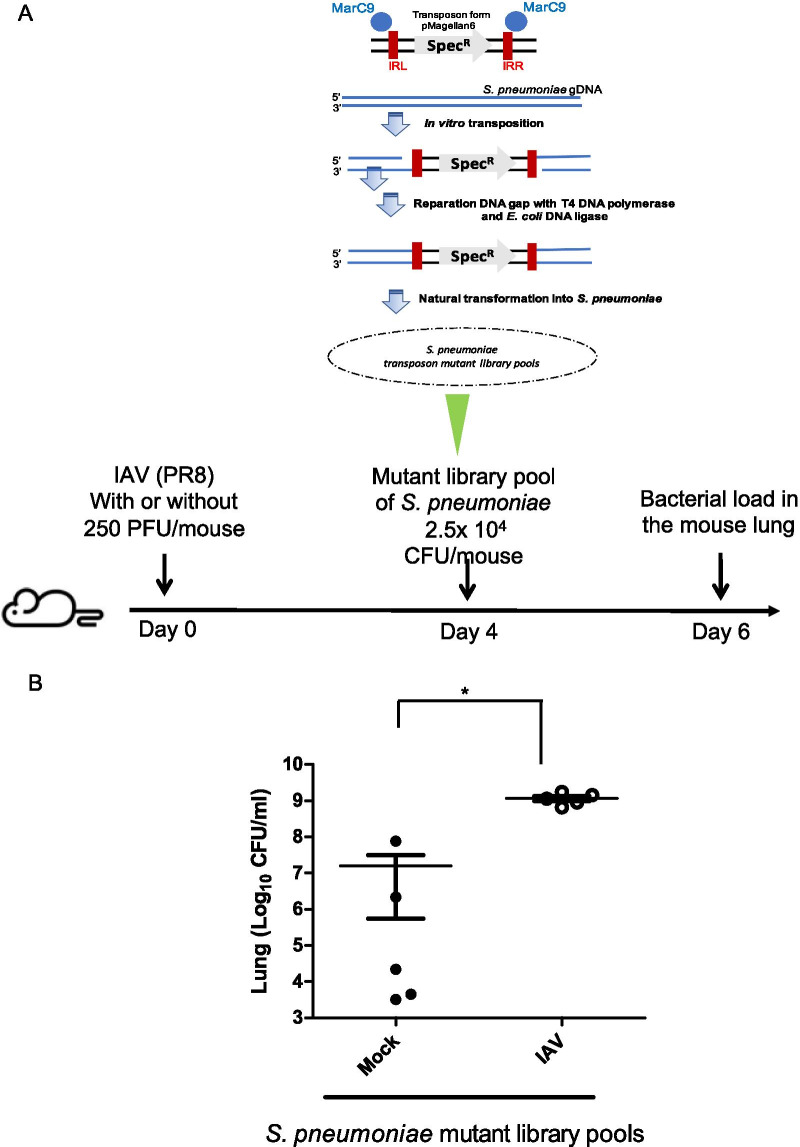
IAV coinfection increased the total bacterial burdens of the mice. **A** Schematic *S. pneumoniae* serotype 3 mutant library construction and experimental design of mouse infection model. Intranasal challenge of 8- to 10-week-old female BALA/c mice, with or without inoculation of 250 PFUs RP8 influenza virus for 4 days. Fifty microliters of *S. pneumoniae* serotype 3 mutant (2.5 × 10^4^ CFU) library pools were used for intranasal inoculation. **B** The mice were sacrificed 2 days post bacterial infection and the bacterial burdens of the mouse lung was calculated via plate counting (n = 5 in each group). Each symbol represents the bacterial burdens value for an individual animal. Mice pre-challenged with influenza viruses RP8 displayed an increase in the total bacterial burdens in the lung (*, p < 0.01, Mann–Whitney U test). *IAV* influenza A/PR8 virus, *PFU* plaque-forming units, *CFU* colony-forming units, *Mock* without pre-challenged with IAV

Eight to ten-week-old female BALB/c mice were intranasally inoculated with 250 plaque forming unit (PFU) of PR8 in 50 μL PBS, under light isoflurane anesthesia. On day 4 post influenza IAV infection, bacterial mutant library pool or mutants of *S. pneumoniae* were intranasally inoculated with 2.5 × 10^4^ − 1 × 10^6^ colony forming unit (CFU) in 50 μL PBS, under light isoflurane anesthesia (Fig. 1A). One or two days post bacterial infection, the mice were euthanized via decapitation after administering anesthesia using isoflurane as per guidelines issued by the Institutional Animal Care and Use Committee (IACUC) of Chang Gung Memorial Hospital. The lungs were removed, rinsed with PBS, and homogenized. Lung homogenates were serially diluted and plated on blood agar, to determine the number of viable pneumococci. The mouse lung with or without IAV infection was homogenized in PBS and centrifuged using benchtop centrifuges, following which the supernatant of the mouse lung was collected and stored at − 80 °C until use. The mice were monitored daily for disease manifestation. Mice with severe sickness were euthanized humanely, in accordance with the guidelines provided by the institutional ethical committee and protocols approved by the IACUC of the Chang Gung Memorial Hospital. All experiments were approved by the local ethical committee for animal research (approval number: 2017072001). All animals were housed in an animal facility at 22 °C, with a relative humidity of 55%, in a 12 h light/12 h dark cycle, with sterile tap water and food available ad libitum.

### Fitness calculation of the *S. pneumoniae* mutant library in a mouse model

Sample preparation for Illumina sequencing and single gene fitness calculation of transposon mutant library of *S. pneumoniae* were performed as previously described [17]. After sequencing, 20-bp reads were mapped to *S. pneumoniae* genomes (accession number: JAFLNB000000000, Taian-S3) using the program Bowtie v.1.2 [20]. Only reads mapping to single sites and insertions with two or more reads at one time point were included in the analysis. For the input (before mice challenge) and output (after mice challenge) treatments, the number of reads at each transposon insertion location was recorded and analyzed.

### Construction of deletion mutants and complementation strain of *S. pneumoniae*

Target gene deletion mutants were constructed as previously described [21]. The *ribA*, *zmpA*, *prtA*, and putative aminotransferase gene (*PA*) deletion mutants (S3-Δ*ribA*, S3-Δ*zmpA*, S3-Δ*prtA*, and S3-Δ*PA*, respectively) were generated by replacing the gene with a spectinomycin resistance gene cassette, as described below, using primers listed in Table 1. Briefly, coding regions and flanking fragments for the four *genes of interest* (*goi*) from the clinical isolate serotype 3 strain, Taian-S3, were amplified using PCR with the following primer pairs: *goi*-up-F/*goi*-dn-R for *goi* (as mentioned above: *ribA*, *zmpA*, *prtA*, and *PA*). The resulting PCR products were cloned into the pJET2.1 plasmid (Thermo Fisher Scientific). The coding regions of the *goi* were removed using inverse PCR (iPCR) with the primer pairs: *goi*-inverse-F/*goi*-inverse-R and ligated to a PCR-amplified spectinomycin antibiotic cassette (spec-F/spec-R) from the pDL278 plasmid, to create a deletion construct vector. This vector was transformed into *S. pneumoniae* Taian-S3 using competence stimulating peptide-1 (CSP-1), and the transformants were selected using spectinomycin. To construct a *PA* gene complementation strain, a single copy of the *PA* gene was inserted into the non-coding region between SPNOXC16740 and SPNOXC16750 (homologs to OXC141; accession number: FQ312027.1) as described below. First, a 2055-bp fragment of the insertion site was PCR-amplified using the primer pair 12133-F/12136-R and cloned into pJET2.1, to create p12133-12136::pJET. Next, the promoter sequence of the spectinomycin (*Pspec*) gene was PCR-amplified from the pDL278 plasmid, the *PA* gene was PCR-amplified from Taian-S3, and the chloramphenicol (*cat*) antibiotic cassette sequence was PCR-amplified from the pKO3-Km plasmid. These three PCR-amplified products were fused together using an overlapping PCR method, with the primers listed in Table 1. The PCR products were digested using EcoRI and sub-cloned into the EcoRI site of the p12133-12136::pJET plasmid, to generate the complementation vector p12133-*Pspec*-*PA*-*cat-*12136::pJET. The complementation strain (S3-Δ*PA*::*PA*) was created by transforming the p12133-*Pspec*-*PA*-*cat-*12136::pJET plasmid into the S3-Δ*PA* strain using CSP-1 and selecting for chloramphenicol.

**Table 1:** Primers and plasmids used in this study

Primer name	Sequence (5’ to 3’)	Purpose
ribA-flank-F	GTATCATATGCGATACAC	Δ*ribA*
ribA-flank-R	GAGCTGGCTATCATGATG	
ribA-inverse-F	TGACTGATTATCCTTTCTGC	Δ*ribA* (iPCR)
ribA-inverse-R	ATGAACACTTATGAAGGTAATTTAG	
zmpA-flank-FF	CTCAGAAAAAGGAAAAAATC	Δ*zmpA*
zmpA-flank-FR	TCCTAATAGTAAACATATTCCTCCTTGAAA	
zmpA-flank-RF	TTTCAAGGAGGAATATGTTTACTATTAGGA	
zmpA-flank-RR	CACGACTGAGAGATAATTCTAA	
zmpA-inverse-F	TATTCCTCCTTGAAATAAAATTTATATATG	Δ*zmpA* (iPCR)
zmpA-inverse-R	TGTTTACTATTAGGAAATAAAG	
Putative aminotransferase-flank-F	TGACACCGCTTCATTTTTCA	Δ*PA*
Putative aminotransferase-flank-R	CTTTCCTTGTTGACCCACAG	
Putative aminotransferase-inverse-F	CTTTGTCTTATCCTTCTAAG	Δ*PA* (iPCR)
Putative aminotransferase-inverse-R	AAATCCAGCCTTCTAGGAG	
prtA-flank-FF	GAAGGAACCACGACACTGC	Δ*prtA*
prtA-flank-FR	TCTATAGCTTTTGTCTTTAATTCCTTACAT	
prtA-flank-RF	ATGTAAGGAATTAAAGACAAAAGCTATAGA	
prtA-flank-RR	CATCAACTGAGCCAGAATATTTG	
prtA-inverse-F	TTTAATTCCTTACATATTTATTTAAAC	Δ *prtA* (iPCR)
prtA-inverse-R	GACAAAAGCTATAGAAAAAAATG	
Psepc-F_EcoRI^a^	CT**GAATTC**GAAGATCGATTTTCGTTCGTG	Promoter region sequence of spectinomycin
Pspec-R_PLP-F_OL	ATCATATTTTCCCATTATTTTGATTAGTACC	
PA-F_Pspec-R_OL	GGTACTAATCAAAATAATGGGAAAATATGAT	Putative aminotransferase gene
PA-R_CAT-F_OL	TTCAATCTATATCACTTAACGTTTGGCAAA	
CAT-F_PLP-R_OL	TTTGCCAAACGTTAAGTGATATAGATTGA	Chloramphenicol
CAT-R_EcoRI^a^	GT**GAATTC**TTATTTATTCAGCAAGTC	
12133-F	TTGCAAGATAAGATTATCCAG	Δ*PA*::*PA*
12136-R	TTAAACGGATATTCTTTAGAG	

Plasmid	Features	Reference
pDL278	*spec*^r^; *E. coli*-Streptococcous shuttle vector	[47]
pJET1.2/blunt	Cloning vector	Thermo Fisher

^a^Boldface letter indicates EcoRI cutting site

### Metabolomics analysis

Bacteria were grown until OD_600_ = 0.5−0.8, following which the number of bacteria was adjusted to 10^8^ CFU, and extracted using extract solution (methanol: acetonitrile: ddH_2_O = 2:2:1 with 2 mg/L 2-chloro-l-phenylalanine), then homogenized using 5 μm glass beads. The sample was centrifuged and vacuum dried then reconstituted with 50% acetonitrile and storage at − 80 °C until for analysis. Each sample (10 μL) was injected into a vanquish-focused ultra-high-performance liquid chromatography (UHPLC) system (Thermo Orbitrap Elite) coupled with an Orbitrap Elite Mass Spectrometer (Thermo Fisher Scientific) using electrospray ionization. Please see Additional file 1 for more detail methods.

### Statistical analysis

Prism 5.0 (GraphPad) was used for statistical analysis. Comparisons of CFU recovered from murine lung were made using Mann–Whitney U test. Data have been presented as mean ± standard deviation (SD) of more than three independent experiments. Differences with p ≤ 0.05 were considered statistically significant.

## Results

### Genome-wide fitness screening of *S. pneumoniae* mutants using a murine IAV coinfection model

At 2 days post-infection with *S. pneumoniae*, the mice were sacrificed, and the total bacterial burden in the mouse lung was calculated. The results showed that pre-challenge with IAV significantly increased the total bacterial burden in the mouse lung (Fig. 1B, p = 0.0079). To identify the bacterial genes that contribute to IAV and *S. pneumoniae* coinfection, we generated a transposon insertion mutant library comprising approximately 2000 individual mutants and inoculated the mutant library pool into a mouse model with or without IAV infection. After calculating fitness, genes (size > 1 kb and a read count ≥ 8) with a fitness score of 0 were designated as required under a given in vivo condition. We identified 24 genes that were required for both bacterial infection and IAV coinfection, and six that were not required for both bacterial infection and IAV coinfection. No gene was solely essential for single or coinfection (Additional file 2). Among the 24 genes that were required for both bacterial infection and IAV coinfection, eight genes encoded hydrolases, five genes encoded transferases, three genes were related to transport, and two genes encoded hypothetical proteins. The remaining genes were related to carbohydrate metabolic processes, lyases, helicases, oxidoreductases, protein kinases, and transcription regulators. One of the identified genes, *nanA*, was previously reported to have a synergistic effect on *S. pneumoniae* coinfection with IAV [11, 22]. Four of the 24 genes [*ribA* (SPNOXC02060 homolog), *zmpA* (SPNOXC10390 homolog), *prtA* (SPNOXC05890 homolog), and *PA* (SPNOXC13360 homolog)] were selected, and deletion mutants of these genes were created for further studies (Table 2 and Additional file 2).

**Table 2:** Four genes that were selected to construct deletion mutants

Number	Gene/product name	Putative function	Relative reference^a^
1	r*ibA*	3,4-Dihydroxy-2-butanone 4-phosphate synthase / GTP cyclohydrolase II	[48, 49]
2	*prtA*	C5a peptidase precursor	[23, 50]
3	*zmpA*	IgA-specific metalloendopeptidase	[51, 52]
4	Putative aminotransferase	PLP-dependent beta-cystathionase / Maltose regulon repressor	

^a^Previously studies to be associated with the virulence of *S. pneumoniae*

### Validation of the roles of the selected bacterial genes in a coinfection mouse model

**Fig. 2:**
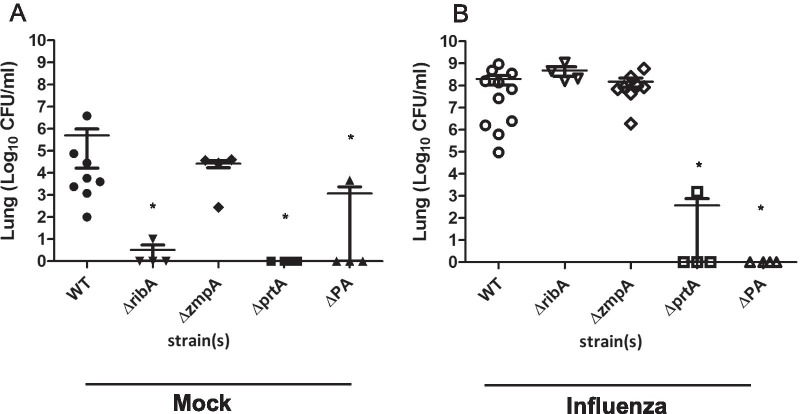
Validation of the role of selected bacterial genes in the coinfection mouse model. Intranasal challenge of 8 to 10-week-old female BALB/c mice (n = 4−11 in each group), without (**A**; solid symbol) or with (**B**; open symbol) inoculation of 250 PFUs IAV for four days. Fifty microliters of *S. pneumoniae* Taian-S3 *goi* deletion mutant (2.5 × 10^4^ CFU) was used for intranasal inoculation. Two days post bacterial infection, the mice were sacrificed and the bacterial burdens of the mouse lung was calculated via plate counting. Each symbol represents the bacterial burdens value of an individual animal (*, p < 0.01, Mann–Whitney U test compare to WT). *CFU* colony forming unit, *Goi* gene of interest, *Mock* without pre-challenged with IAV

The four selected genes were individually deleted in the clinical serotype 3 strain Taian-S3, which is the parental strain used for mutant library construction, by precise replacement of each coding region with the spectinomycin antibiotic resistance sequence. The four deletion mutants, S3-Δ*ribA*, S3-Δ*zmpA*, S3-Δ*prtA*, and S3-Δ*PA*, were tested in a mouse model with or without IAV infection. The *ribA* gene was only required for colonization of mouse lung in the bacterial infection alone (Fig. 2A, B). Two genes (*prtA* and *PA*) were required for colonization of the mouse lung in the single and coinfection models (Fig. 2A, B) as predicted from the genome-wide fitness analysis. PrtA was previously reportedly associated with *S. pneumoniae* virulence [23], which is consistent with the results of our study. Thus, this association was not investigated further.

### Putative aminotransferase is involved in the cystine and methionine metabolic pathway and regulates the growth of *S. pneumoniae*

**Fig. 3:**
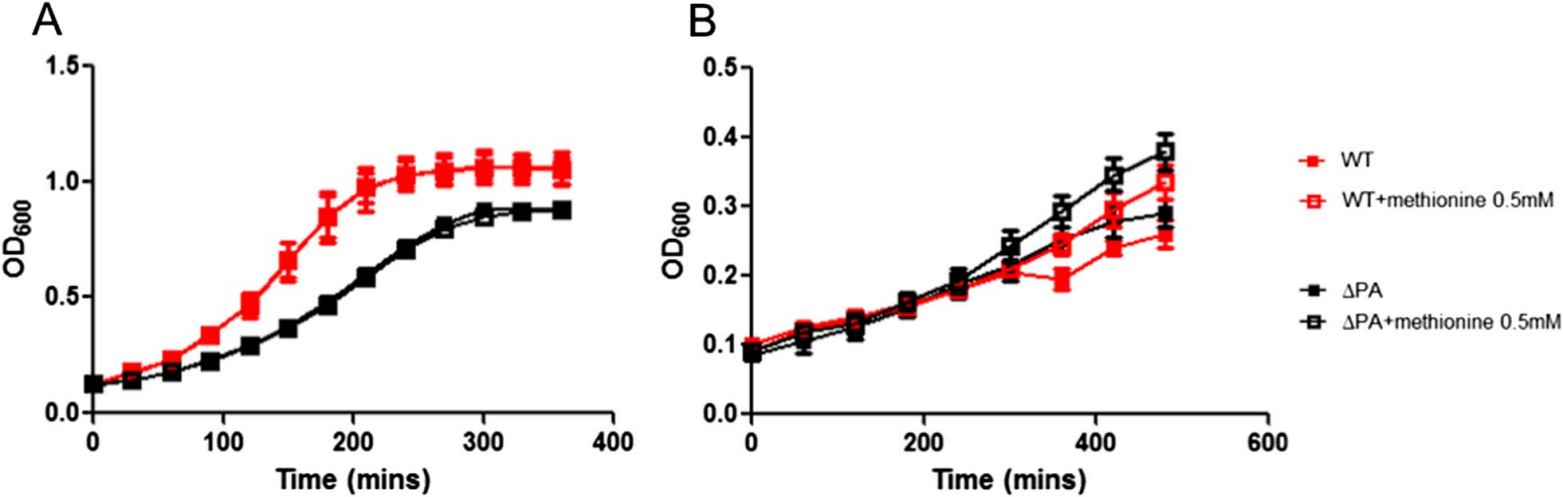
Methionine supplementation restored the growth of putative aminotransferase gene deletion mutant and increased the growth of the wild-type strain. Bacterial growth kinetics (OD_600_) of *S. pneumoniae* Taian-S3 wild-type strain (red, WT) and putative aminotransferase gene deletion mutant (black, ΔPA), with (empty square) or without (filled square) 0.5 mM methionine supplementation, were assessed every 30 min in THY medium (**A**) and every 60 min in CDM medium (**B**). Data are shown as mean ± standard deviation (SD) from three to five independent experiments. *CDM* chemically defined medium

Analysis of the growth kinetics of the four deletion mutants showed that the growth of S3-Δ*PA* was significantly slower than the kinetics of the other mutants, which grew similar to the wild-type Taian-S3 strain (Additional file 1: Fig. S1A). A *PA* gene-complemented strain (S3-Δ*PA*::*PA*) showed restored growth kinetics similar to that of the wild-type strain (Additional file 1: Fig. S1B). PA from Taian-S3 shares 39% amino acid identity with the *Bacillus subtilis* PatB polypeptide. PatB is a class I pyridoxal phosphate (PLP)-dependent aminotransferase [24] that participates in the synthesis of methionine and cystine [24, 25]. To investigate the effect of methionine supplementation on growth kinetics, the growth of the S3-wild-type strain and the S3-Δ*PA* mutant were compared in both THY complete medium and CDM medium with restricted methionine content. In THY, there was no growth difference between the S3-wild-type strain and the S3-Δ*PA* mutant with or without methionine (Fig. 3A). However, in CDM, the growth of both the S3-wild-type strain and S3-Δ*PA* mutant were increased by the addition of methionine (Fig. 3B). We speculated that, in a nutrient-restricted environment, the addition of methionine can improve growth. We then comprehensively investigated the differences in the metabolite composition of the S3-wild-type strain and S3-Δ*PA* mutant using an untargeted mass spectrometry (MS)-based metabolomics analysis. In the negative charge metabolite analysis, 26 metabolites were found to be significantly different between the S3-wild-type strain and S3-Δ*PA* mutant. In the positive charge metabolite analysis, 51 metabolites were found to be significantly different between the S3-wild-type strain and the S3-Δ*PA* mutant (Additional file 3). Most of the metabolites could not be annotated (MS1 and MS2), and only a few could be identified by their metabolite IDs in the KEGG database. Among those that were identified, such as L-aspartic acid (KEGG: C00049) and glutathione (KEGG: C00051), which are involved in the cystine and methionine metabolic pathways, were higher in the S3-Δ*PA* mutant than in the S3-wild-type strain.

### Lung colonization is impaired in putative aminotransferase gene-deficient pneumococci and is partially restored upon IAV coinfection

**Fig. 4:**
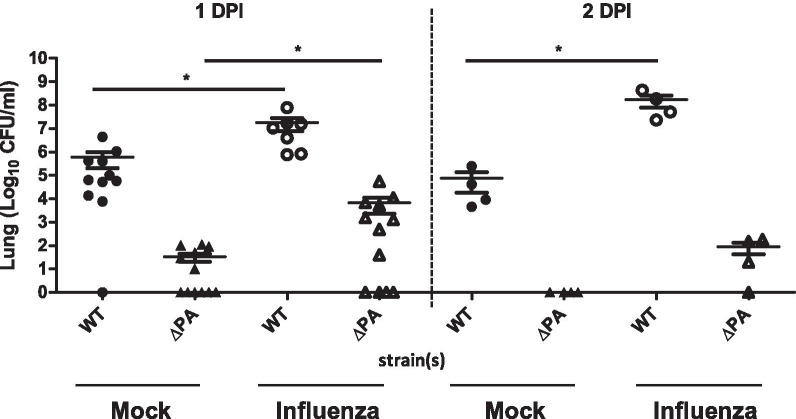
Bacterial colonization is impaired in putative aminotransferase gene-deficient pneumococci and is partially restored upon IAV coinfection. Mice (n = 4−12 in each group) were intranasally infected with or without IAV, followed 4 days later by intranasal inoculation with 1 × 10^6^ CFU of *S. pneumoniae* putative aminotransferase gene deletion mutant (S3-Δ*PA*) or wild-type strain (S3-WT). After one or two days post bacterial infection, the mice were sacrificed and the bacterial burden in the mouse lung was assessed via plate counting. Each symbol represents the bacterial burdens value for an individual animal (*, p < 0.01, Mann–Whitney U test compare to mock-treated). *CFU* colony forming unit, *DPI* days post-bacterial infection, *Mock* without pre-challenged with IAV

To assess the role of the *PA* gene in lung colonization by *S. pneumoniae* during coinfection with influenza, mice were infected intranasally with *S. pneumoniae* Taian-S3 wild type or its isogenic *PA* deletion mutant (S3-Δ*PA*) at 4-day post IAV infection. Since the growth kinetics of S3-Δ*PA* were slower, to better discriminate the difference in the bacterial burden in the mouse lung, we increased the bacterial infection dosage from 2.5 × 10^4^ CFU/mouse to 1 × 10^6^ CFU/mouse. The mice were sacrificed at one or two days post bacterial infection. The results showed that the bacterial burden in the lung of S3-Δ*PA*-infected mice was significantly lower than that of S3-wild-type strain-infected mice under single infection conditions (Fig. 4). Coinfection with IAV significantly increased the lung colonization density of both the S3-wild-type strain and S3-Δ*PA* mutant when compared to that with bacterial infection alone (Fig. 4). Based on this finding together with increased bacterial growth upon methionine addition in vitro, we speculated that the improved growth of S3-Δ*PA* mutant and S3-wild-type strain after IAV infection might be due to changes in the nutrients/metabolites present in the mouse lung upon IAV infection.

### IAV-infected murine lung homogenate increases *S. pneumoniae* growth

**Fig. 5:**
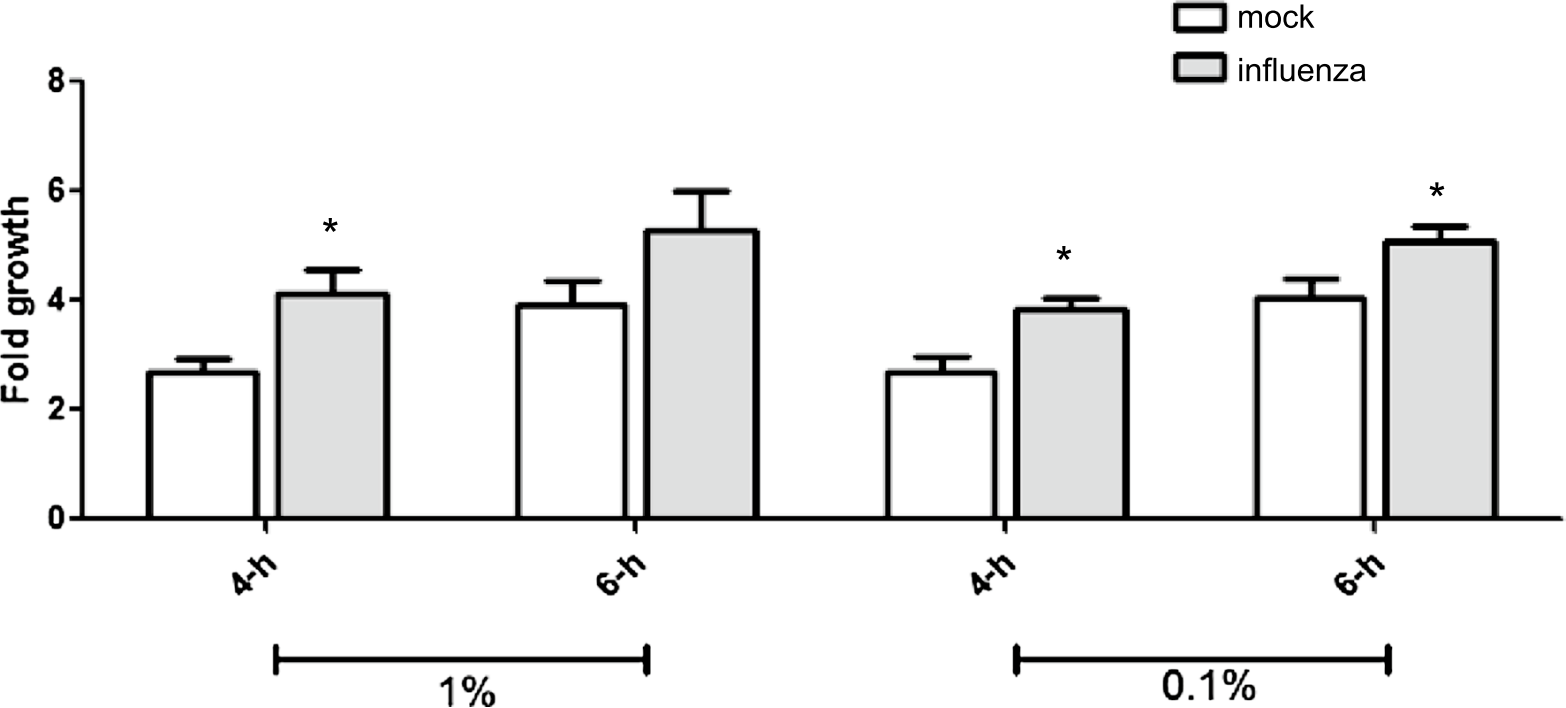
IAV-infected mouse lung can increase *S. pneumoinae* bacterial growth. IAV-treated or mock-treated (without IAV infection) mouse lung was homogenized and added (at 1% or 0.1% of total volume) in CDM. Bacterial number of *S. pneumoniae* Taian-S3 wild-type strain upon murine lung homogeneous extract supplementation was determined via plate counting at 0 h, 2 h, and 4 h, followed by calculation of the fold change in bacterial number. Statistical analysis was performed using Mann–Whitney U test (*, p = 0.05 compare to mock-treated). Data are shown as the mean ± standard deviation (SD) from three independent experiments. *Mock* without pre-challenged with IAV

Supplementation with methionine promoted bacterial growth in vitro, and coinfection with IAV increased the bacterial burden in mouse lung. Next, we sought to examine whether the mouse lung (post-IAV infection) could directly increase pneumococci, to rule out the host immune system. We added a crude lung homogenate extracted from mice with or without IAV infection to a *S. pneumoniae* culture in CDM medium (with restricted nutrient content) and assessed the fold change in bacterial growth. As expected, the fold change in bacterial growth of the S3-wild-type strain was higher in cultures with homogenate from virus-infected mice than in cultures with homogenate from mock-treated (without IAV infection) mice (Fig. 5). In summary, we demonstrated that a crude extract from mouse lung post-IAV infection promoted bacterial growth, which is coherence with the observed increase in bacterial burden in mouse lung after IAV infection.

## Discussion

Serious secondary bacterial pneumonia frequently occurs after influenza infection. *S. pneumoniae* is the major pathogen causing secondary bacterial pneumonia post-influenza infection [1, 26]. Primary influenza infection can modulate the host immune response by increasing either the anti-inflammatory or pro-inflammatory response, reducing bacteria clearance and leading to poor outcomes from secondary bacterial pneumonia [27, 28]. In addition, virus-induced cytotoxicity leads to lung epithelial cell damage resulting in cell attachment side exposure, thus increasing bacterial adhesion and physical barrier dysfunction in the airway [1, 26]. These changes lead to an increase in host susceptibility to secondary infections. Viral-bacterial synergism also contributes to serious secondary bacterial infections [11, 18]. Neuraminidase, which is expressed in both influenza and pneumococcus, possesses sialidase activity and contribute to release of sialic acid from host airway mucins [10, 11]. Therefore, pneumococcus can utilize more host sialylated substrates as a nutrient source to promote bacterial growth [11] and biofilm formation [11, 22] after influenza infection. However, the detailed mechanisms and bacterial virulence factors that contribute to the synergistic effect between influenza and pneumococcus in coinfections are not completely understood. To determine if there are other genes or synergism mechanisms that are involved in this viral-bacterial interaction, a systematic genome-wide bacterial fitness screening of a *S. pneumoniae* mutant library pool was used. The screen identified the putative aminotransferase gene, which is involved in the cystine and methionine biosynthetic pathway. Based on our analyses performed in vitro and in vivo for the *PA* gene-deficient mutant of a serotype 3 strain, we hypothesized that the increase in *S. pneumoniae* growth after influenza infection may result due to changes in the host metabolome, and the utilization of these metabolites may promote bacterial fitness after influenza infection.

Studies have shown that influenza infection dynamically changes the metabolome in serum, the lungs, and BALF of mice [29]. In previous studies in animals, the common trends in the metabolome post-influenza infection include alterations in amino acids and related molecules, in select lipids, and in some nucleosides, nucleotides, and analogs; increases in carbohydrates and related molecules; and decreases in mannitol, myo-inositol, and glyceric acid [29–32]. Metabolite changes in the host post virus infection might reflect the consequences of viral manipulation of the host metabolism to favor the production of new viral particles and modulation of pro-inflammatory and anti-inflammatory metabolites that contribute to disease pathogenesis. Moreover, infection with other viruses, such as respiratory syncytial virus (RSV) and severe acute respiratory syndrome coronavirus 2 (SARS-CoV-2), can also alter the host metabolome [33–35]. Infections with these respiratory viruses are also often followed by severe secondary bacterial pneumonia [36, 37]. The metabolome changes accompanying influenza infection that affect metabolic pathways are closely related to pro- and anti-inflammatory cytokine expression levels. For example, amino acid metabolites are correlated with IFN-γ and IL-6 expression [30]. In addition, the metabolic reprogramming associated with influenza infection in adipose tissue and preadipocytes promotes influenza virus replication [38]. Therefore, obesity, which changes the T-cell metabolome, is a risk factor for increased mortality from influenza infection [39]. Accordingly, the effects of the metabolome changes that occur with influenza infection, which may increase the incidence of secondary bacterial infection, are worthy of further study. One study revealed that capillary leakage, with the efflux of nutrients, especially glucose, into the alveolar space after influenza infection promotes pneumococcal growth [40]. The results of the current study were consistent with previously research showing that the metabolites of the host lung were changed after influenza infection. In addition, our findings showed that supplementation of a bacterial culture with a crude homogenate from influenza-infected lungs increased *S. pneumoniae* growth also revealed that the metabolome changes in the mouse lung could directly promote bacterial growth.

Bacteria can increase their fitness in the host niche by producing metabolites, such as essential amino acids, to promote colonization [19], virulence [18, 41], biofilm expansion [42], and rapid growth [40]. Methionine it is one of the scarce amino acid in physiological fluids but its importance cannot be underestimated. Methionine biosynthetic genes are essential for the full virulence of many bacteria, including *Haemophilus parasuis* [43], *Brucella melitensis* [44], *Salmonella enterica* [45], and *S. pneumoniae* [41]. Consistent with previous findings, the results of this study showed that the poor growth rate of a *PA* gene-deficient mutant could be restored by supplementation with methionine, and pre-infection with influenza A virus could also increase the bacterial growth of the *PA* mutant.

In this study, only 30 genes were identified in the genome-wide screening as being related to infection. One identified gene, *nanA*, was previously reported to have a synergistic effect with IAV [11, 22]. Therefore, the results of our screen were consistent with data from other studies, and we expect that our mutant library screen results are still representative of the viral-bacterial interaction. Although fewer genes were identified in this study, we still found the *PA* gene in our mutant library pool and revealed the importance of metabolome changes after influenza infection in bacterial growth.

## Conclusions

Because of the recent increases in antimicrobial-resistant non-pneumococcal conjugate vaccine (non-PCV) *S. pneumoniae* types and new subtypes of influenza that continue to cause global pandemics [46], influenza and *S. pneumoniae* coinfection remains a significant public health concern. Specific bacterial virulence factors that contribute to bacterial-viral interaction may be useful therapeutic targets to control or prevent secondary bacterial pneumonia after influenza infection. The results of the study indicate that bacterial metabolites that cannot be synthesized by the host may serve as potential drug targets to combat secondary bacterial pneumonia induced by serotype 3 pneumococci upon influenza infection.

## Supplementary Information

**Additional file 1.** Supplemental appendix for Materials & Methods and Results.

**Additional file 2.** Thirty genes that were identified in the genome-wide screening method in this study, and gene annotation of Taian-S3.

**Additional file 3.** Metabolome comparison of S3-wild-type strain and S3-ΔPA mutant.

## Data Availability

The Whole Genome Shotgun project of *S. pneumoniae* serotype 3 clinical strain, Taian-S3, during this study were deposited at DDBJ/ENA/GenBank under the accession JAFLNB000000000. The version described in this paper is version JAFLNB010000000.

## Abbreviations

CDM: Chemically defined medium
CFU: Colony forming unit
Goi: Genes of interest
IACUC: Institutional Animal Care and Use Committee
IAV: Influenza A virus
LB: Luria broth
MDCK: Madin-Darby Canine Kidney
PA: Putative aminotransferase
PCV13: 13-Valent pneumococcal conjugate vaccine
PFU: Plaque forming unit
THY: Todd–Hewitt broth supplemented with 0.5% yeast extract
UHPLC: Ultra-high-performance liquid chromatography
