# Supplementary material for: Bacterial factors required for Streptococcus pneumoniae coinfection with influenza A virus
Source: J Biomed Sci. 2021 Aug 27;28:60. doi: 10.1186/s12929-021-00756-0 (PMC8395381; doi:10.1186/s12929-021-00756-0)
Supplement: Supplementary file 1 — Additional file 1. Supplemental appendix for Materials & Methods and Results. [file 12929_2021_756_MOESM1_ESM.docx]

**Appendix**

**Bacterial factors required for *Streptococcus pneumoniae* coinfection with influenza A virus**

**Yi-Yin Chen^1^, Ching-Tai Huang^2^, Shiao-Wen Li^3^, Yi-Jiun Pan^4^, Tzu-Lung Lin^5^, Ya-Yu Huang^1^, Ting-Hsuan Li^1^, Yu-Ching Yang^1^, Yu-Nong Gong^6,7^, Yu-Chia Hsieh^1*^**

***Correspondence:**

Yu-Chia Hsieh

Department of Paediatrics, Linkou Chang Gung Memorial Hospital, No5, Fuxing Street, Guishan District, Taoyuan City 333, Taiwan.

E-mail addresses: yuchiahsieh@gmail.com

Telephone: +886-3-3281200 ext. 8231

Fax: +883-3-3286210

**Supplementary Methods**

**Purification of maltose binding protein−Himar1 Mariner MarC9 transposase**

Ten times dilution of overnight-cultured of *E. coli* pMalC9 strain (a gift from Dr. Tim van Opijnen) in 80 mL LB (with 100 mg/L ampicillin) was cultured at 37°C with aeration until the OD_600_ reached approximately 0.5. Following this, the cells were induced with 0.3 mM Isopropyl β- d-1-thiogalactopyranoside (IPTG) at 37°C, with aeration for 2 h. After centrifugation for 10 min at 8,000 × *g*, 4°C, the cells were resuspended in 10 mL cold column buffer (20 mM Tris-HCl, pH 7.4; 200 mM NaCl; 1 mM EDTA; 1× complete EDTA-free protease inhibitor cocktail). The cell pellet was lysed using sonication and the clarified lysate was transferred to a new 15 mL centrifuge tube on ice, and discard the pelleted cell debris by centrifuging for 10 min at 18,000 × *g*, 4°C. The cell lysate was incubated with 1 mL transposase wash buffer-washed amylose resin (New England Biolabs) at 4°C on a rocking platform for 1 h to bind the maltose-binding transposase protein, MarC9. The resin was washed more than five times with 2 mL cold transposase washing buffer (20 mM Tris-HCl, pH 7.4; 200 mM NaCl; 1 mM EDTA; 2 mM DTT; 10% (v/v) glycerol; 1× complete EDTA-free protease inhibitor cocktail), following which the maltose-binding MarC9 was eluted in cold transposase elution buffer (20 mM Tris-HCl, pH 7.4; 200 mM NaCl; 1 mM EDTA; 2 mM DTT; 10% (v/v) glycerol; 10 mM maltose; 1× complete EDTA-free protease inhibitor cocktail). The purified MarC9 transposase was dispensed into ~10 μL aliquots and stored at −70°C [1].

**Construction of a *S. pneumoniae* transposon mutant library by in vitro transposition with the transposase MarC9**

The pMagellan6 plasmid DNA contained the mini-transposon magellan6 (a derivative of the Himar1 Mariner transposon), which encodes for the spectinomycin resistance marker cassette (Spec^R^). As illustrated in Figure 1, in vitro transposition of 1 μg *S. pneumoniae* genomic DNA with 1 μg of the plasmid pMagellan6 was carried out using 0.5 μL purified MarC9 at 30°C for 1 h. The DNA was precipitated using sodium acetate, washed with ethanol, and dissolved in 12.5 μL water. The transposon junctions were repaired by incubating genomic DNA with T4 DNA polymerase at 12°C for 20 min and with *E. coli* DNA ligase at 16°C overnight. Linear fragments of genomic DNA containing magellan6 insertions were transformed into *S. pneumoniae* using competence-stimulating peptide-1 (CSP-1), to yield a magellan6-inserted *S. pneumoniae* mutant library.

***S. pneumoniae* transposon library sample preparation for Illumina sequencing post IAV coinfection**

The bacteria from the lungs of superinfected (virus + bacteria) or single-infected (bacterial only) mice were collected, and bacterial genomic DNA was extracted from them using the QIAamp® DNA Mini Kit (Qiagen). Genomic DNA of S. pneumoniae was digested with MmeI at 37°C for 2.5 h, followed by dephosphorylation using alkaline phosphatase, calf intestinal (CIP; New England Biolabs), at 37°C for 1 h. The MmeI-digested genomic DNA was extracted with phenol/chloroform, precipitated with 95% ethanol, and then resuspended in 25 μL of distilled water. Twenty-five microliters of repurified-DNA was ligated with 0.2 mM of Illumina sequencing-annealed adapter (22) using T4 DNA ligase, at 16°C overnight. The ligation mixture was prepared using the following PCR reaction: 1 cycle at 95°C for 3 min, 18-22 cycles at 95°C for 10 s, 55°C for 25 s, 72°C for 5 s, and 1 cycle at 72°C for 10 min, to amplify the 120 bp target region using the primer pairs, P1_M6_MmeI and Gex PCR Primer 2, and PfuUltra DNA polymerase (Agilent). PCR products were separated on a 2% agarose gel and the 120 bp band was excised under low UV light, purified using a QIAquick® Gel Extraction Kit (Qiagen), eluted in 50 μL distilled water, and analyzed using Illumina sequencing.

**Metabolomics analysis**

Bacteria were grown until OD_600_=0.5−0.8, following which the number of bacteria was adjusted to 108 CFU, and washed once with PBS, after which the remaining PBS was removed. After the addition of 300 μL of extract solution (methanol: acetonitrile: ddH_2_O=2:2:1 with 2 mg/L 2-chloro-L-phenylalanine), the samples were homogenized using 5 μm glass beads, sonicated for 10 min in an ice-water bath, and incubated for 1-h at −20°C, to precipitate the proteins. The sample was then centrifuged at 13,000 rpm for 15 min at 4°C. The resulting supernatant was transferred to a fresh 1.5 mL tube and vacuum dried using SpeedVac SPD111V (Thermo Fisher Scientific). The pellets were reconstituted with 50% acetonitrile and transferred to a new glass vial for analysis. The quality control (QC) sample was prepared by mixing an equal aliquot of the supernatant from all the samples. Each sample (10 μL) was injected into a vanquish-focused ultra-high-performance liquid chromatography (UHPLC) system (Thermo Orbitrap Elite) coupled with an Orbitrap Elite Mass Spectrometer (Thermo Fisher Scientific) using electrospray ionization. UHPLC parameters were set as follows: A 2.1 × 100 mm Acquity BEH 1.7 μm C18 column (Waters Corporation) was used. The column oven temperature was set at 40°C. The binary mobile phase consisted of deionized water containing 0.1% formic acid as solvent A and LC-MS grade acetonitrile with 0.1% formic acid as solvent B. The flow rate was 0.25 mL/min, with a linear gradient elution over 15 min. For the first minute, solvent B percentage was held at 0%, linearly increased to 100% for the next 7 min, kept constant for 3 min, and finally returned to 0% in 1 min. To avoid any carry-over effect, there was one blank injection post every sample injection, and one QC injection post every five sample injections, for peak area normalization. Mass spectrometry (MS) data were collected in positive mode with a default data-dependent acquisition method: one MS full scan performed in profile mode at 60,000 resolution, followed by 10 data-dependent MS2 scans at 15,000 resolution. The mass scan range was set from 70 to 1000 m/z. A normalized collision energy (NCE) of 25 was used. The spray voltage was 3.5 kV and the capillary temperature was set at 280°C. The sheath gas was set at 30 arbitrary units, and the auxiliary gas was set at 5 arbitrary units.

**Supplementary Figures**

**Fig. S1:** Growth kinetics of *goi* from the clinical isolate serotype 3 strain, Taian-S3. (A) Growth curve, as measured in terms of OD_600_, of Taian-S3 wild-type strain and its isogenic gene deletion mutants for *ribA*, *zmpA*, *prtA*, and *PA*. (B) Growth curve, as measured in terms of OD_600_, of Taian-S3 wild-type strain (S3-WT; ●), putative aminotransferase gene deletion mutant (S3-Δ*PA*; ⯅) and its gene complemented mutant (S3-Δ*PA*::*PA*;⯆). Each strain was assessed in triplicate (n=3). Goi, gene of interest; PA, putative aminotransferase

**Supplementary Reference:**

1. van Opijnen T, Lazinski DW, Camilli A. Genome-wide fitness and genetic interactions determined by Tn-seq, a high-throughput massively parallel sequencing method for microorganisms. Curr Protoc Microbiol. 2015;36:1E 3 1-24. https://doi.org/10.1002/9780471729259.mc01e03s36 PMID:25641100.
